# Medial patellofemoral ligament reconstruction: a new technique

**DOI:** 10.1186/1471-2474-8-22

**Published:** 2007-02-28

**Authors:** Michael R Carmont, Nicola Maffulli

**Affiliations:** 1 Department of Trauma and Orthopaedics, University Hospital of North Staffordshire, Keele University School of Medicine, Stoke on Trent, ST7 4QG UK

## Abstract

**Background:**

Primary patellofemoral dislocations are common. In most patients, non-operative management produces satisfactory outcome. If the dislocation recurs after a trial of rehabilitation, operative intervention is considered, with the aim of restoring the soft tissue anatomy to normal. Ninety four percent of patients suffer a tear to the medial patellofemoral ligament (MPFL) following a patellar dislocation.

**Results:**

We describe our transverse patella double tunnel technique to reconstruct the medial patellofemoral ligament using a free autologous gracilis or semitendinous graft.

## Background

Patellofemoral dislocations are common, and tend to occur with as a result of quadriceps contraction across a flexed, valgus knee with the weight bearing tibia externally rotated compared to the femur [1]. The dislocation usually reduces spontaneously or with muscle relaxing drugs.

Once the normal relationship of the patellofemoral joint is restored, patients may begin their rehabilitation. Non operative management is the recommended option following primary patellar dislocation [2,3]. Conservative management focuses on concentric exercises to strengthen the quadriceps, and especially the vastus medialis, to prevent further instability. If the dislocation recurs after a trial of rehabilitation, operative intervention is considered, with the aim of restoring the soft tissue anatomy to normal. Ninety four percent of patients suffer a tear to the medial patellofemoral ligament (MPFL) following a patellar dislocation [4]. These lesions have been classified according to there anatomic location by Nomura [5]. In cadavers, MPFL reconstruction showed a significant reduction in lateral displacement and ligament load compared with medial transfer of the tibial tuberosity [6].

We describe our transverse patella double tunnel technique to reconstruct the medial patellofemoral ligament.

## Results

**Figure 1:**
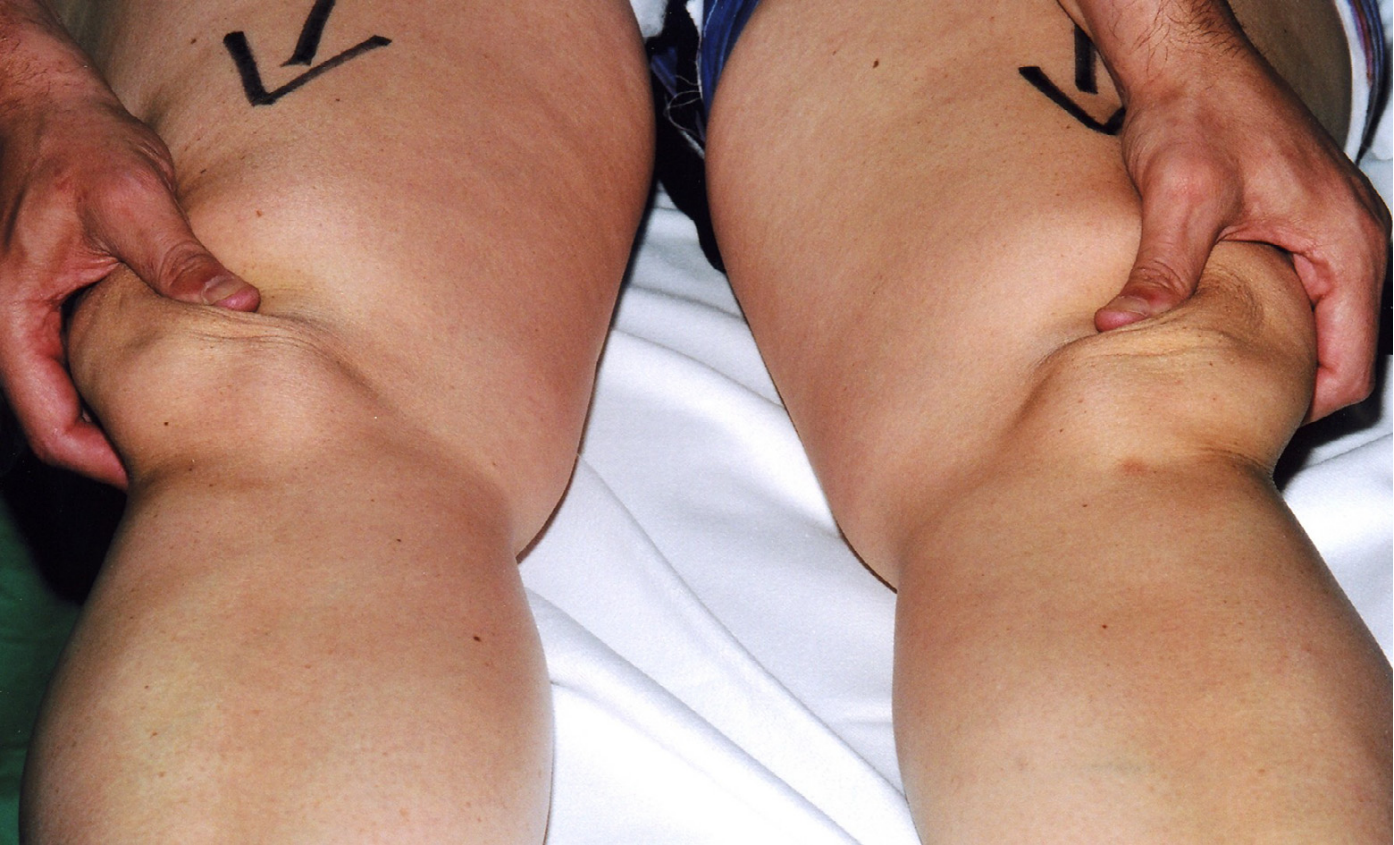
Examination under anaesthesia revealing marked patella instability.

The patient is placed supine, with an above knee tourniquet, following the administration of prophylactic antibiotics (Figure 1). Skin preparation and sterile draping is performed in a standard fashion.

The tendons of semitendinosus and gracilis are harvested in the usual fashion [7,8]. The tendon is debrided of muscle tissue, prepared with a Vicryl locking suture (Ethicon, Edinburgh) on both ends, sized using anterior cruciate ligament (ACL) tunnel sizers, and stored within a moist swab.

**Figure 2:**
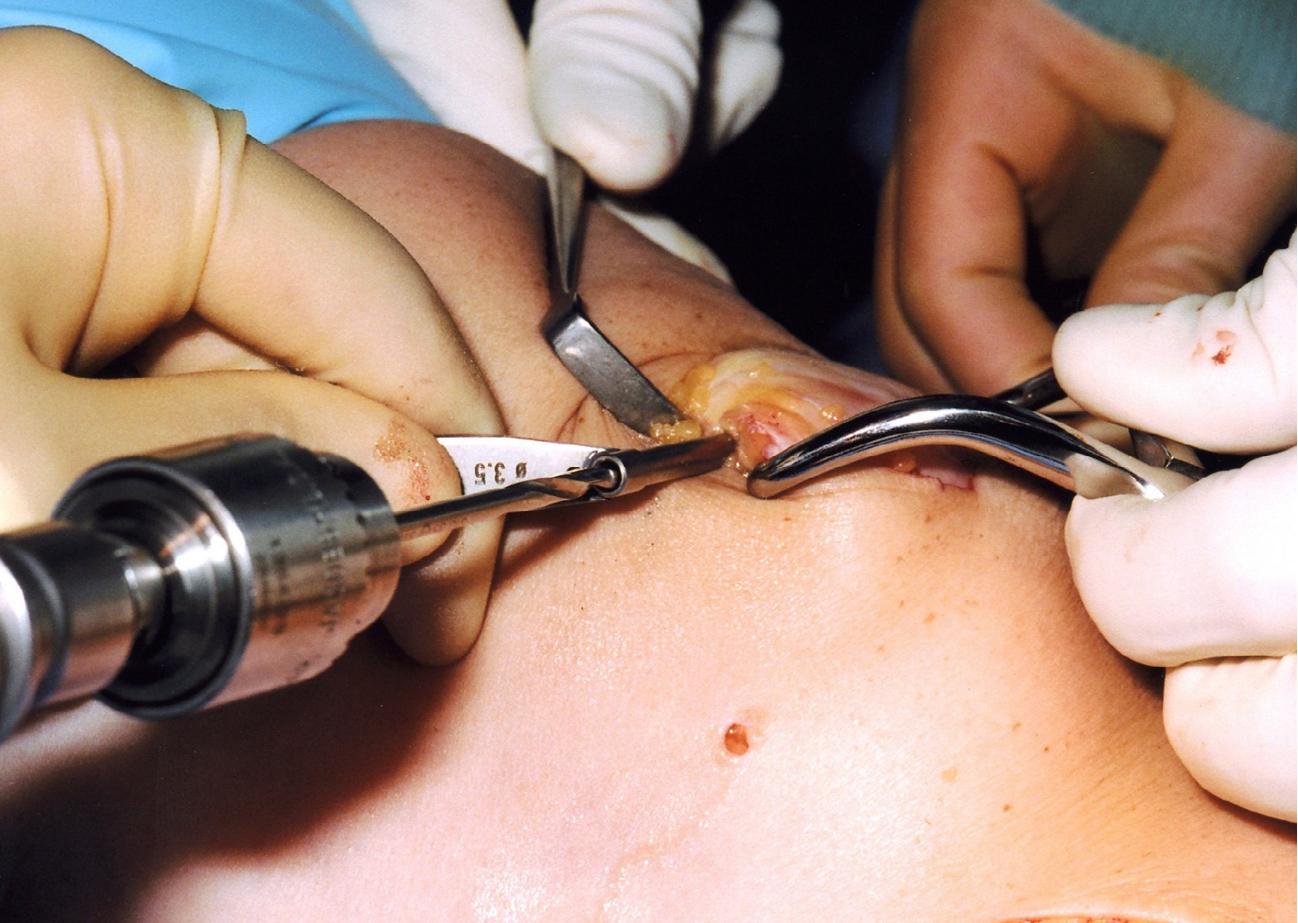
Following medial and lateral parapatellar incisions, the patella is stabilised using a large clamp on the right of the figure. Tunnels are produced by sequential drill holes in the superior half of the patella, 1 cm apart.

**Figure 3:**
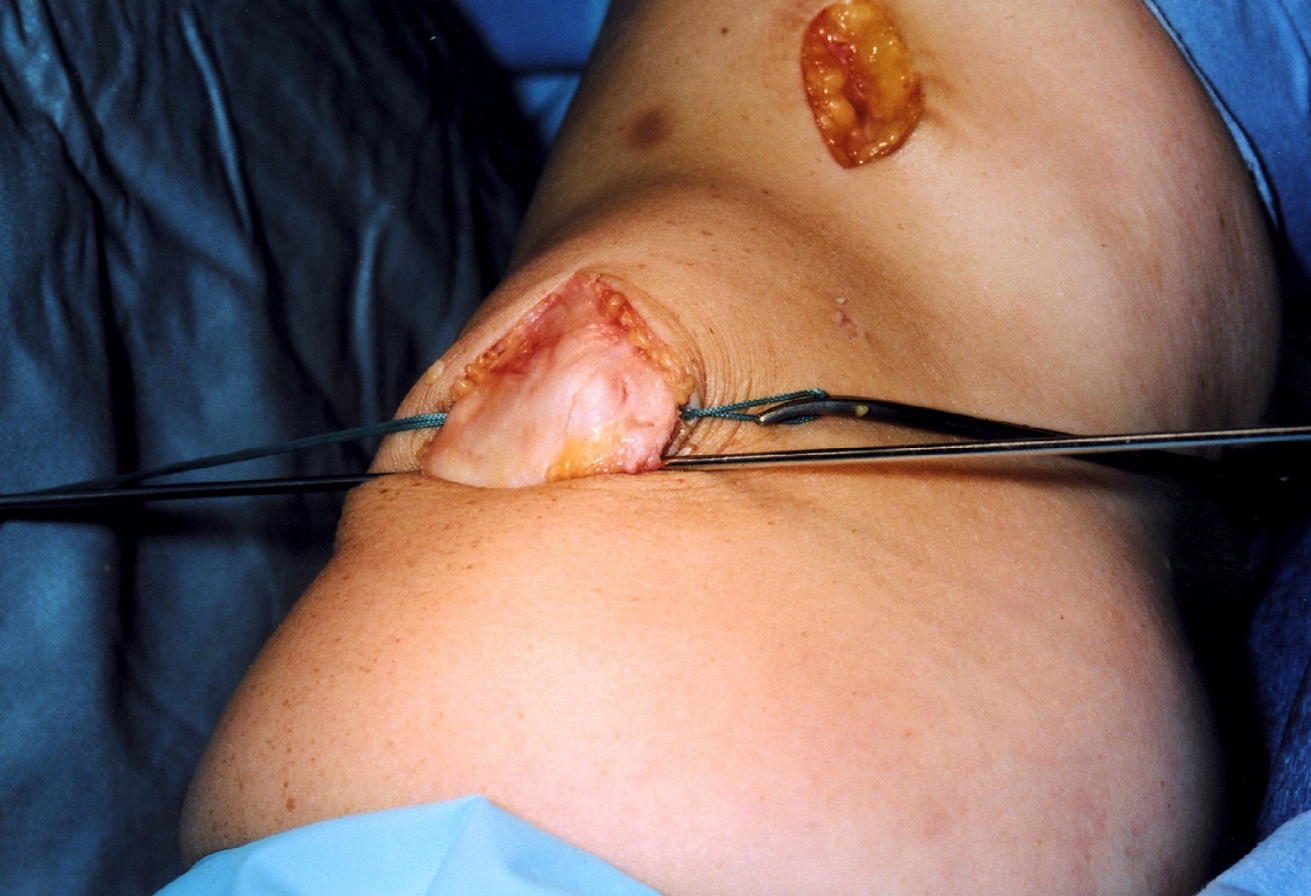
A Beath pin is used to pass a Vicryl loop through the patella tunnels.

**Figure 4:**
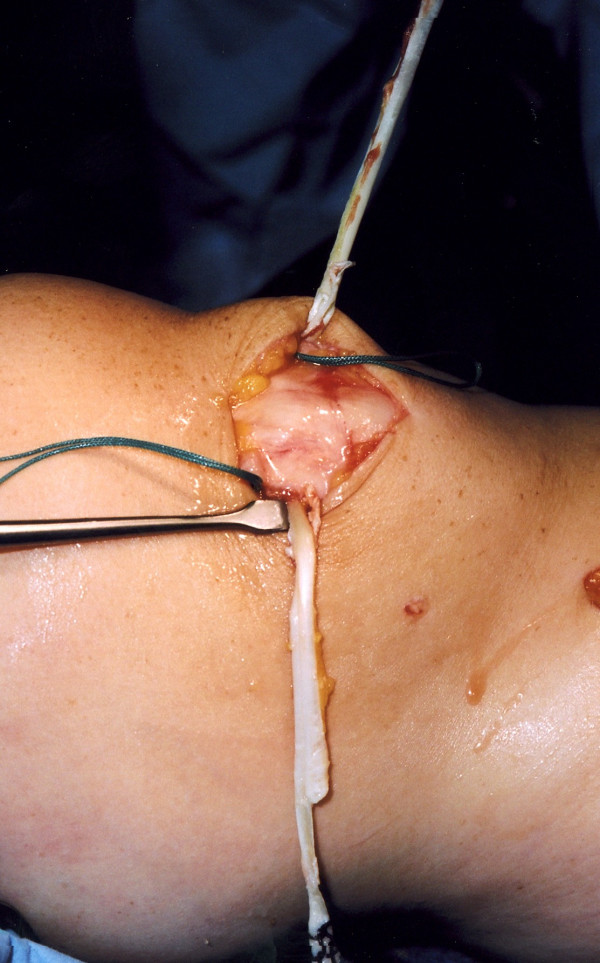
The graft is passed through the tunnels, laterally then medially.

The patella is approached through a 4 cm midline incision. The prepatella fascia is elevated to allow the medial and lateral walls of the patella to be exposed through medial and lateral parapatellar incisions. The inferior portion of the patella is stabilised using large forceps. Using sequential tunnel enlargement with appropriately sized drills, two transverse tunnels are made in the upper third of the patella to accommodate a single thickness of the graft (Figure 2). The tunnels are drilled parallel to one another and 1 cm apart. Using a Beath pin, the graft is threaded through the two transverse tunnels from medial to lateral (Figure 3), and then from lateral to medial (Figure 4) so that the graft forms a loop through the patella. It is easier to advance the graft passing the thinner end first (normally, the portion of the tendon inserting on the pes anserinus) with local anaesthetic jelly (Instillagel, Farco Pharma, Cologne).

**Figure 5:**
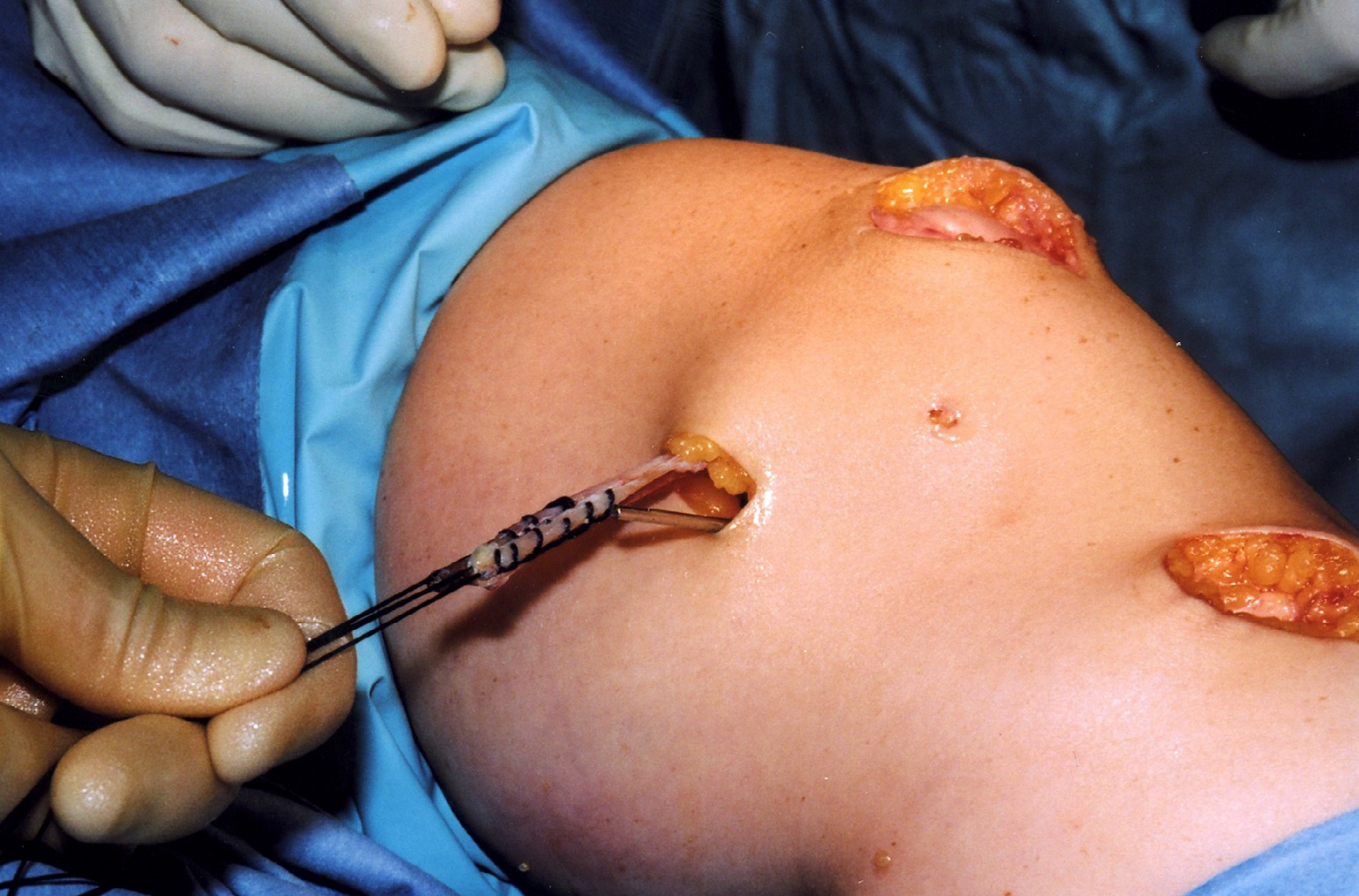
The medial epicondyle is exposed and the Beath pin is placed across the transepicondylar axis and a tunnel is drilled to accommodate and secure both ends of the graft. The graft is passed between the second and third layers of the knee.

Curved blunt forceps are used to develop a plane between the second and third layers of the knee. The graft is passed through the plane between these layers (Figure 5).

**Figure 6:**
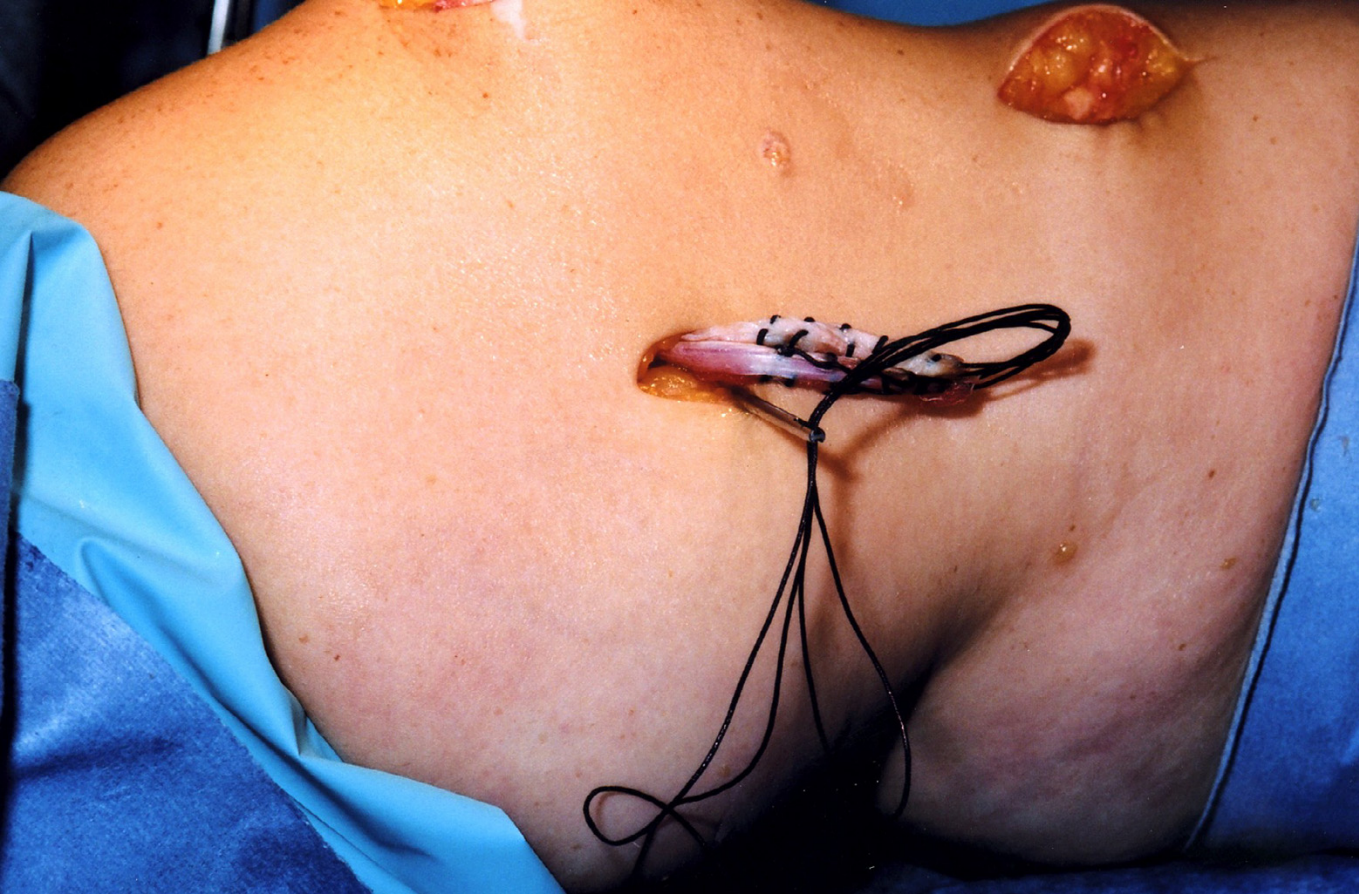
The graft is pulled into the tunnel using Vicryl through the eye of the Beath pin.

**Figure 7:**
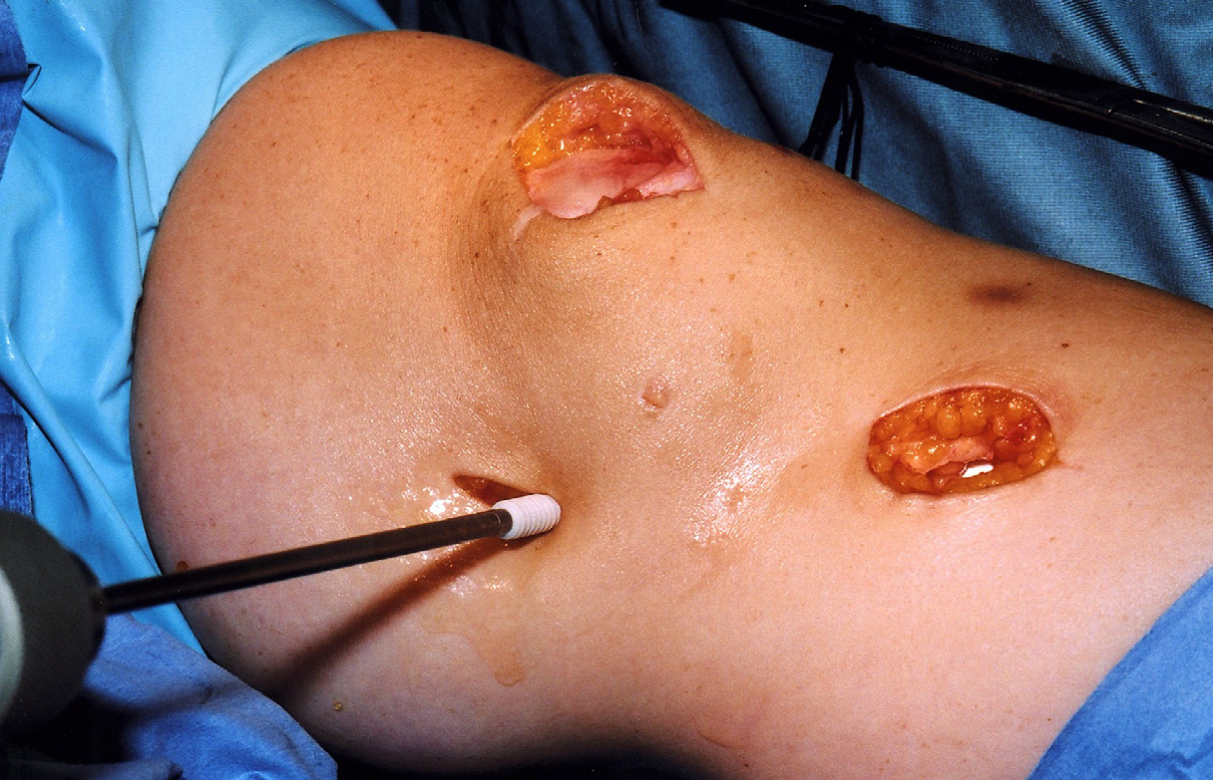
After cycling the knee through a full range of movements to allow graft tension to settle, the graft is secured using an interference fit screw.

**Figure 8:**
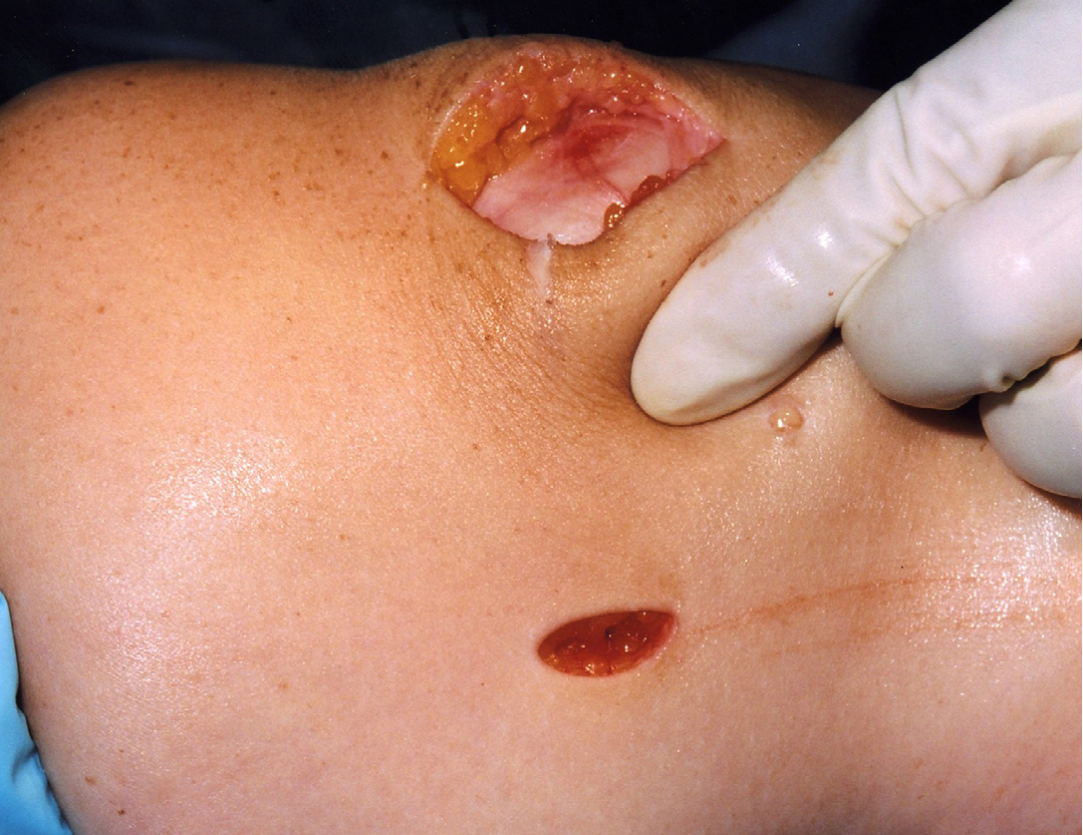
The improved stability of the patella is confirmed.

The medial epicondyle is palpated through the skin and exposed using a 2 cm incison. The Beath pin is then advanced along the transepicondylar axis laterally from the superior aspect of the medial epicondyle. A medial blind tunnel, normally about 3 cm long, is drilled along the guide pin to accommodate a double thickness of graft to an adequate depth to allow optimal graft tension. The Vicryl locking suture is then passed through the transepicondylar axis using the Beath pin, pulling the graft into the medial tunnel, and the patella is positioned in the femoral trochlea (Figure 6). The knee is cycled several times from full flexion to full extension with the graft under tension. In this way, the graft is prestretched to eliminate "give". Both ends of the graft are then secured within the medial epicondyle tunnel using a bioabsorbable interference fit screw (Depuy Mitek, Norwood, MA) (Figure 7, Figure 8) with the knee flexed to 20°. The graft thus acts as a check rein ensuring that the patella is stabilised within the trochlea. In summary, the graft passes through the superior and inferior transverse patella tunnels, forming a loop through the patella, with both ends being secured in the medial epicondyle tunnel.

The lateral and medial retinacula are sutured back to the patella using Vicryl, with further closure of subcutaneous tissues and skin. Routine dressings, bandages and a cricket pad splint are applied.

Post-operative mobilisation regime consists of full weight bearing in a cricket pad splint. After two weeks, all restrictions are removed and the patient is allowed to return to normal activities over the course of three months.

## Discussion

The medial side of the knee consists of three layers. The first layer consists of the deep or crural fascia forming a layer which invests sartorius but is superficial to gracilis and semitendinosus. The second layer forms the fibres of the superficial medial ligament. Here, anterior fibres pass upwards to blend with the vastus medialis, and posterior fibres run from the patella to insert at the medial epicondyle. The third deep layer forms the capsule of the knee joint. Vertically aligned fibres form the deep layer of the medial ligament or the middle capsular ligament to the mid portion of the medial meniscus and the tibia [9]. The MPFL is 5 to 12 mm wide [10].

The MPFL is the major medial soft tissue restraint preventing lateral displacement of the distal knee extensor mechanism, contributing an average of 53% of the total force [11]. The MPFL is located within the second layer of the knee, and it may have a role in the prevention of lateral excursion of the patella [12]. Desio found similar values of 60% at 20° knee flexion [13]. The ligament has a mean tensile strength of 208 N [14]. The inferolateral to superomedial fibres of the MPFL only change in length by 1.1 mm during knee flexion from 0° to 90° [15]. Patellar tracking is significantly affected by a lateral force in the absence of the MPFL, but returns to normal following reconstruction [16].

Reconstruction techniques include primary repair [17], ligament imbrication [18], reconstruction using autogenous tissue [19-27] or synthetic graft [28,29].

The remainder of the discussion focuses on reconstruction techniques. The semitendinosus and gracilis hamstrings are commonly harvested as grafts for soft tissue reconstructive procedures [7]. Several different methods have been described to reconstruct the MPFL with hamstring graft, and variation also occurs between tunnel placement and graft fixation methods. The graft attachment points of the reconstructed of the MPFL are the superior patella for the lateral attachment and the superior aspect of the medial epicondyle for the medial attachment. Varying attachment points have been described but an area between the medial epicondyle and adductor tubercle is considered optimal [30,31] however this may be difficult to identify in larger knees when using small incisions. Mountney has cadaverically compared different tunnel and anchoring techniques. A tendon graft in a blind tunnel in the femur has a tensile strength of 126 N and a through tunnel tendon graft has a tensile strength of 195 N, not significantly different to the original MPFL [14]. Bioabsorbable interference screw and endobutton-post fixation have comparable strength when used in ACL reconstruction [32].

Ellera Gomes utilised a 2.5 mm horizontal tunnel within the patella, enlarged on the medial side to 3.2 mm for the initial 10 mm. The tendon graft was sutured into the patella tunnel and the medial graft was looped through the adductor magnus tendon and sutured onto itself [33].

Muneta performed MPFL reconstruction on patients with residual instability after medial transfer of the tibial tubercle. The hamstring graft tissue was passed through the second layer of the knee into a 4.5 mm drill hole in the middle of the patella to the centre of the patella. From here, the graft was anchored through an additional 3.2 mm hole to the superficial surface of the bone, being secured with a button. The femoral epicondyle insertion was fixed with a staple [19]. Schock describes a double loop of semitendinosus passed through a single patella tunnel and anchored in place with button and suture material on the patella and a cancellous screw at the epicondyle [20]. Fernandez describes anchoring a single strand hamstring graft with two 2.5 mm drill holes at the end of a 4.5 mm tunnel [21]. In a technique recently described by Farr, a double strand of semitendinosus is used as a graft, with the medial ends sutured together, secured in a medial epicondyle tunnel and the lateral ends separated forming a V shape inserting onto the medial patella [27].

A recent series by Drez reports a 93% improvement in symptoms after MPFL reconstruction for patella instability. The hamstring tendon was doubled, the strands sutured together and then sutured under tension to the superomedial edge of the patella and the medial epicondyle. A second limb of tissue was then sutured to the tibial periosteum just below the joint line [22].

A study comparing the fixation of hamstring graft to the patella reported no difference between suturing to the patella periosteum and using a single tunnel to the centre of the patella [23]. A similar method has recently been described by Nomura, with 66% giving excellent results at 3 years follow up [24].

The quadriceps tendon has been utilised to provide ligament tissue. The superficial layers of the quadriceps tendon have been folded medially, rerouted through the second layer of the knee and attached onto the medial epicondyle [25]. The use of a bone block in addition to quadriceps tendon has also been described [26]. One of the earliest described reconstruction techniques used a polyester ligament passed through a bone tunnel at the midline of the patella [28]. Nomura prospectively reviewed the use of a mesh artificial ligament (Leeds-Keio) and a medial retinacular slip reconstruction, with 96% patients having excellent or good results [29].

One of the important aspects of any ligament reconstruction is the tension of the graft. Computational analysis has shown that small alterations in length and position of the graft can dramatically increase the force and pressure applied to the medial patellofemoral cartilage [31,33]. To try to optimise graft tension, we, with other authors, recommend cycling the knee through its range of motion prior to fixation with the knee flexed at 20°. This aims to remove "give" from the graft prior to fixation. The graft was secured under adequate tension (qualitatively assessed) to act as a check rein, preventing patella subluxation.

Complications include impingement of the graft on the medial femur during flexion [34]. This could be enhanced due to the use of a single thinner graft. In our method, the doubled graft should exert less stress on the femur during flexion because of increased area of contact during movement, thus minimising unpleasant impingement symptoms. Indeed, in more than 30 patients followed for at least 3 years we have never experienced this complication. Another benefit is the reduction of a medial stabilising force over a more natural thickness of tissue. Anatomical studies have shown the graft to be 5–12 mm wide. Single strand techniques utilise hamstrings of about 3.5 mm wide. This can be doubled over to give a thicker ligament, but may cause difficulty with the placement of a larger single tunnel within the patella. A larger patellar tunnel may increase the risk of joint penetration or patellar fracture [23]. Our two tunnel technique allows a wide tendon graft but uses small tunnels, thus minimising these potential complications. Also as the tunnels traverse the entire width of the patella this technique may influence patella tilt.

We have described our technique of medial patellofemoral ligament reconstruction. This double tunnel technique allows a wider ligament comprising of a double thickness of hamstring to be reconstructed, minimising graft impingement, without increasing the risks of patellar fracture compared to a single tunnel technique.

## Competing interests

The author(s) declare that they have no competing interests.

## Authors' contributions

MC wrote the paper and performed the literature search. NM is the senior author who has developed the surgical technique and has proof read and rewritten the paper. Both authors have read and approved the final manuscript.

## Pre-publication history

The pre-publication history for this paper can be accessed here:
